# Crania Americana; or a Comparative View of the Skulls of Various Aboriginal Nations of North and South America; to Which Is Prefixed an Essay on the Varieties of the Human Species

**Published:** 1840-07

**Authors:** 


### REVIEWS.

---

Art. V.—*Crania Americana; or a comparative view of the Skulls of various Aboriginal nations of North and South America: To which is prefixed an Essay on the Varieties of the Human Species. Illustrated by seventy-eight plates, and a colored map.* By Samuel George Morton, M.D., Professor of Anatomy in the Medical Department of Pennsylvania College at Philadelphia; member of the Academy of Natural Sciences of Philadelphia; of the American Philosophical Society; of the Historical Society of Pennsylvania; of the Boston Society of Natural History, &c., &c. Philadelphia; J. Dobson, Chesnut street. London; Lumpkin, Marshall & Co., 1839. Letter press, p. 296, folio.

This volume is the product of a bold and somewhat original enterprise in the highest branch of natural science—we mean anthropology. And of that enterprise, wide and varied as are its scope and bearings, high its purposes, and important its aim, it is not unworthy. Nor does any thing perhaps contribute more to its attractiveness and value, than its simplicity and unpretendingness—its freedom we mean from self-glorifying theories and doctrines, and more especially from iron dogmas, and air-woven hypotheses. It consists almost exclusively of an immense body of facts, no inconsiderable portion of them new, collected in a laborious and protracted course of reading, observation, and multiform research, and neither misinterpreted, perverted, nor misapplied, in subservience to the influence of preconceived notions. Instead of itself broaching or even propagating crude opinions, or giving existence and shape to any thing like a premature and ephemeral system, it aims at the ascertainment and confirmation of elementary truths, out of which, as imperishable materials, substantial and lasting opinions and systems may be hereafter constructed.

Dr. Morton, in the great work before us, has neither so restricted his scheme, as to fit it in all respects to the study of man, as *an individual,* nor so expanded it, as to suit it especially to that of mankind *as a single body.* He has given to it rather an *ethnographical* character—adapted it, we mean, to researches into the conditions, analogies, differences, and relative standing of mankind, as divided into *tribes, families,* and *nations.* And, if we mistake not, he has had, as a physiologist, the discernment to perceive, and the firmness and independence, as a man and a writer, to select and avow, the veritable ground, out of which those differences of human condition and standing essentially arise. In plain terms; without professing to be a phrenologist, and apparently, if not really, unconscious that he was so—and certainly without any predetermined intention to be either an advocate or an opponent of the new scheme of mental philosophy—under these circumstances, our author's production is as strictly phrenological, as any of the publications of Gall or his followers. And, as will appear hereafter, the entire and fast multiplying host of his facts, as far as they bear on the science, (and from their inordinate number, they might be called "legion") are expressly confirmatory of it. In no respect could its warmest advocates change them for the better, or even wish them changed—we mean as regards their phrenological bearing. In all time to come therefore, the "Crania

Americana" will be referred to, by enlightened anthropologists, in proof and illustration of the truth and importance of the discoveries of Gall. But to come into closer contact with our subject.

That the organic structure of the human body, in its aggregate capacity, is composed of a number of subordinate structures, is known to *every one.* And to the *anatomist* and *physiologist* it is known, that, of these subordinates, the nervous structure, including the brain and spinal cord, holds the highest rank. Though several of the others are as essential to its existence and the efficiency of its condition, as it is to theirs, it is notwithstanding so far the master tissue, as to control the others, and so far predominant in its general influence, as to give to man his native supremacy and fitness for empire, and place him at the head of the animal creation. In all the other structures which enter into his organism, the more perfect of the inferior animals are the equals of man, and in some of them his superiors. In the character of his nervous tissue alone he is pre-eminent. And, in full acknowledgment and demonstration of that pre-eminence, the phrenologist pronounces and proves the brain, which is but a portion of the nervous tissue, to be the *organ of the mind*—to be that instrument, or rather *array of instruments,* (for it is strikingly multiplex in its structure as well as in its functions,) by which alone the mind executes its purposes, and manifests its power. Nor, however different may have been the case, but a few years ago, does any *physiologist* now, who is worthy of the *title*, venture to question the truth of this position.

Such then is the foundation, (the supremacy we mean of the nervous tissue,) on which phrenology rests; and on the same foundation rests essentially the work we are examining; and from that consideration arise, in an eminent degree, its interest and value. Wherefore? The answer is plain, and easily rendered. It is the difference in the size, form, and general character of the brain, the great nervous centre, which constitutes the leading and most important differences between the several races and varieties of the human family. And to that source does our author virtually ascribe them. True, he refers also to the distinctions created between men by difference in size of body, complexion, hair, features, and personal configuration. These however, he justly considers as but minor points in the production of dissimilitudes. For that difference, which, being itself the chief one, imparts a broad and abiding distinction of constitution and character, he looks not to the blood-vessels, muscles, or bones, nor yet to the viscera of the abdomen or thorax, (though they also are important elements) but to the volume, form, and condition of the brain, as manifested by the size and configuration of the head. Hence the title of his book—"Crania Americana"—tantamount, in its import, to Cerebra Americana.

But in collecting his materials for the volume before us, Dr. Morton did not confine his draughts to the aboriginal tribes and nations of America. Far from it. He framed and filled them under the influence of a more catholic spirit, and with less restricted views, and addressed them to sources much more extended, and more abundant in means. Conformably to this arrangement, he commences his work with an "Introductory Essay on the Varieties of the Human Species," in which he has manifested, for an *American* writer engaged in the active and arduous duties of a profession peculiarly burdened by toils and interruptions, an amount of reading and application, labor and research, exceedingly unusual, if not unprecedented, and rarely equalled, and perhaps never exceeded on any given subject, and under similar circumstances, by a writer of any country. He has judiciously done what every author should feel himself bound to do, from respect toward himself, as well as toward the public—gone to his work with a proud ambition, and a determined resolution to attain and master all the knowledge accessible to him, in relation to the subject he is preparing to handle. By no other mode of proceeding can an author, be his talents and *general* attainments what they may, do full justice to himself or his theme, or produce a work worthy in all respects of public favor.

It is not unimportant to remark, and recommend to remembrance (because it may tend to the prevention of cavils and censures, as respects the "Essay" we are examining,) that the expression, "Varieties of the *Human Species,*" virtually involves a belief, if it does not amount to an avowal of it, in the Mosaic doctrine of the *unity* of man.

Our author, with the habitual correctness of judgment which characterizes him, has adopted that division of the GREAT COMMUNITY OF MAN, which, though not perhaps perfect, we are inclined to think the best, because it is liable to fewest objections. The division is nearly the same with that supported, if not originally devised, by Professor Blumenbach, and classes mankind under five *races;* the *Caucasian;* the *Mongolian;* the *Malay;* the *American;* and the *Ethiopian.* These races our author himself divides into twenty-two families, in the following order:

"I.—THE CAUCASIAN RACE.

1. *The Caucasian Family.*
2. *The Germanic Family.*
3. *The Celtic Family.*
4. *The Arabian Family.*
5. *The Lybian Family.*

6. *The Nilotic Family.*
7. *The Indostanic Family.*

"II.—The Mongolian Race.

8. *The Mongul-Tartar Family.*
9. *The Turkish Family.*
10. *The Chinese Family.*
11. *The Indo-Chinese Family.*
12. *The Polar Family.*

"III.—The Malay Race.

13. *The Malay Family.*
14. *The Polynesian Family.*

"IV.—The American Race.

15. *The American Family.*
16. *The Toltecan Family.*

"V.—The Ethiopian Race.

17. *The Negro Family.*
18. *The Caffrarian Family.*
19. *The Hottentot Family.*
20. *The Oceanic-Negro Family.*
21. *The Australian Family.*
22. *The Alforian Family.*"

Such is our author's division of man into *races* and *families*; and of the distinguishing and characteristic traits of each division, he gives us a brief but graphical description. On his details however it is not our present purpose to dwell; because neither our time nor our space permits us to do so. Notices of his results are all we must attempt. And even they must be limited and few. We could not make them full and circumstantial, without writing an entire *treatise*, instead of an article for a periodical work. Nor would it be possible for us to endue with either much interest or usefulness, any thing we could say of our author's details respecting man in Europe,

Asia, Africa, or Polynesia. The reason is plain. His work neither contains, nor was designed to contain any drawings of the "Crania" of the "races" and "families" which form the population of those parts of the globe. We have therefore no source of instruction, phrenological or philosophical, either to enlighten ourselves, or to enable us to enlighten others, on the subject of their intellect, morals, fitness for civilization, or general native character and developments. All we have of information respecting them is historical or narrative, or some limited description of person and countenance. And we need hardly say, that from such premises no substantial or instructive inferences can be drawn, as respects either their intellectual or moral endowments, or their prevailing propensities — nothing in fact to throw any clear and satisfactory light on the constitution of their minds.

As respects the American race and families however the case is different. Of them our author has laid before us correct delineations of the "Crania," tantamount to delineations of the *brains* themselves, which necessarily corresponded to the crania, of about *fifty* different tribes, varieties, or nations. And those skulls thus represented to us, when correctly interpreted, speak the language of nature and truth, as regards the mental endowments of the tribes and individuals to whom they belonged.

Are we asked, on what particular mental attributes of their owners those figures of skulls are calculated to throw light? We reply, on every attribute, provided they are thoroughly understood; but more especially on every leading attribute, fitted for the elevation or depression of a people, by giving them more or less of animal propensity, and of general mental power and character. They disclose, for example, the comparative amount of *native* intellect possessed by those who wore them; the comparative amount of native morality, sociability, and domesticity; the comparative amount of native pride, independence, and love of liberty, self-government, and sway over others; and also the native amount of animality and passion, manifested in sensual appetite, vindictiveness, cruelty, bloodshed, and war. Nor is this all.

The configuration of the skull and brain discloses likewise something of the different modes in which different tribes and individuals wreak their personal vengeance, or conduct themselves in war—whether by a bold and open attack by day, or by ambush and skulking stratagem, in the night. And when two tribes or nations nearly equal in numbers engage in war, the comparative size and figure of their skulls, foretell in language sufficiently intelligible, and which ought not to be listened to with incredulity or disregard, to which side victory and conquest are likely to incline. They are evidences of the possession or destitution of war-like qualities.

That these sentiments will be received with distrust by many, and perhaps entirely rejected by more of our readers, we are prepared to believe. Nor is the cause of this unknown to us. It is the want of an acquaintance with the principles of the new scheme of mental philosophy. For with those principles the sentiments just uttered are in perfect accordance. They are in harmony with the admitted physiological fact, that the brain and nerves are the master tissue in the organization of our bodies; that they control, strenghten, and direct the other tissues; and that, other things being equal, the greater the amount of cerebral matter individuals possess, they will, whether acting alone, or in union with their fellows, prove the more powerful, efficient, and successful in their enterprizes. And the more likely they will be to become civil rulers in peace, and temporary victors, and permanent conquerors in periods of strife. Of course, on the contrary, a tribe or nation, whose skulls and brains are comparatively diminutive are, in consequence of that defect, the less able to defend themselves, and the more liable to be vanquished and enslaved, or exterminated, in war.

Such we say are the grounds, on which the sentiments just expressed are founded. And not only are they in unison with the science of phrenology; by many of the plates in the "Crania Americana" they are abundantly sustained. And of the following points respecting human character, those plates furnish also, in equal abundance, matter of satisfactory illustration and proof.—Other things being alike, the more the animal organs of the brain predominate in a tribe or community over the moral, religious, and reflecting ones, the more ignorant and vindictive, blood-thirsty and cruel in war, and other forms of conflict and punishment, whether public or private, will that community show itself.—The larger in a tribe or nation the organs of self-esteem, love of approbation, conscientiousness, and firmness are, other things being equal, the more difficult of conquest will that tribe be found, and the more certainly will it prefer extermination to slavery.—Once more. Other things being the same, large moral, religious, and reflecting organs facilitate the civilization of a people—and the reverse; and when the moral and religious organs are large in a community, and the intellectual, animal and semi-animal ones small, that community will submit to bondage, rather than to extermination, and perhaps even rather than to banishment from its native soil. To illustrate and prove these positions, by materials derived from our author's "Crania."

Those plates demonstrate, that in the brains of what the writer calls the "American family," which constitutes chiefly the aboriginal people of *North* America, the reflecting, moral, and religious organs are comparatively small, and the animal and semi-animal ones proportionably large. And the experience of more than two centuries has abundantly evinced, that that "family," as a body, can be neither civilized nor actually conquered and enslaved; but that their ultimate extinction is an event which is approaching, and whose accomplishment nothing earthly can prevent. This is true of the entire family, on account of the general similarity of their organization, the animal and semi-animal portions of their brains being preponderant. But in some branches of the family this is more signally the case than in others. And their propensities and characters correspond, with great exactness, to their cerebral developments. Thus the animality and semi-animality of the Charibs are immense, while their moral, religious, and reflecting organs are correspondingly small. And they are, beyond all other tribes, wild and indomitable, ferocious and sanguinary. Fierce, warlike and unyielding, rather than submit to conquest and slavery, or to any form of civil restraint, they covet extermination, which is nearly accomplished. Of the Huron tribe, whose cerebral developments are in no small degree analogous, the same may be said. They have refused to yield, have fought desperately, and practised every form of cruelty, and are nearly extinct. With an organization and development of brain, and a condition of mind not dissimilar, the Seminoles are pursuing at present a course of warfare, which, if not abandoned, must lead in the end to a like result.

Possessed of brains, as appears from their skulls, more liberally supplied with moral, religious, and intellectual organs, the Creeks, Choctaws, and Cherokees, though brave, warlike, and proverbially artful, have shown themselves less inexorably cruel, and less brutally devoted to havoc and blood. They are even reported by a few persons, and believed by many, to exhibit faint glimmerings of an approach to civilization. This however is but groundless rumor. Even of the Cherokees, believed to be the more cultivated of the three tribes, this may be affirmed. The "*full-bloods*" among them are degraded savages. It is the "*half-bloods*" alone, and other mixtures, more or less approaching full Caucasianism (and their number is small) that exhibit any positive traits of civilization and improvement. The chieftain Ross was almost white; Opotheoholo had also much Caucasian blood in him; and the inventor of the celebrated Cherokee alphabet was the son of a Scotchman. And the cerebral developments of the two first named of these, whom we saw in Washington, corresponded sufficiently with their talents and characters. Nor indeed can the Scotch-Cherokee be correctly pronounced the inventor of the alphabet in question. He was only the fortunate receiver, from a Caucasian of a plain and practical suggestion, of which the alphabet was ultimately the product. The stories so widely and zealously circulated, proclaiming the Cherokees an industrious, civilized, agricultural *people*, are rank fabrications, designed no doubt for selfish and party purposes. Considered as a tribe or nation, nineteen out of twenty of them, and perhaps even a larger proportion, are indolent, degraded, and miserable savages. And, instead of having property, as they are asserted to have, a majority of them, probably not much less than that just stated, are pennyless wretches, in a much worse condition than Caucasian paupers.

Another tribe well worthy of being noticed in this place, on account of the light it sheds on the connexion between cerebral development, mind, and character, is the *Araucanian.* That people inhabit one of the Chilian provinces, toward the southern extreme of South America, and in the excellent development of their brain, as well as in their amount of native intellect, improvability, vigor, and general efficiency of character, stand at the head of the American "RACE," the Mexicans and Peruvians, of former times, in some respects excepted.

In size and shape, the skull of the Araucanian makes a nearer approach to the skull of the Caucasian, than that of any other variety of the aborigines of America. And so does the individual himself in quickness, strength, and compass of mind, and in the energy, firmness, and efficiency of his action, whether he be engaged in hunting or war, or in any other less exciting and perilous pursuit. In the organs especially of Self-esteem, Firmness, Conscientiousness, Combativeness and Love of Approbation, his developments are large. *Hence* his lofty pride and spirit of independence, with his devotedness to a life of liberty, and his resolution to maintain those priviliges at every hazard and every cost, have never yielded under any form of adversity, or degree of suffering. For perhaps a century and a half his unconquerable daring, and determination to be free have led him to sustain a ceaseless war with the Spaniards on his borders; and his resources of intellect, but little inferior to those of his foe, and disciplined into skill by trial and experience, have enabled him to do so with uniform success. Still however do his boundless pride, and his reckless and ungovernable aversion from the slightest check on his licentious freedom, coupled with a deficiency of reflectiveness and moral feeling, prevent him from submitting to the mild and salutary restraint of civilization. With all his qualifications therefore for a different state of life, he is still a savage. And he is so, as the result of his cerebral development, which renders him intolerant of the control of law, and makes him resolve, like Christian, in Byron's "Island," "to live and die, the fearless and the free."

When attentively studied and thoroughly understood in their nature and relations, the whole case and condition of the Mexicans and Peruvians, ancient and modern (for *they* have their ancient and modern epochs as distinctly marked, and contrasted in as broad and bold relief, as the Europeans and Asiatics)—the case and condition of these nations, when fully and correctly comprehended, present one of the most extraordinary spectacles in the history of man. And, as a moral problem, its solution is as difficult, not to say impracticable, as its aspect as a phenomenon is singular and interesting. Though reiterated attempts to that effect have been made by philosophers the most distinguished for their general knowledge and powers of research, no approach that can be called even *seemingly* successful, has yet been made toward causes competent to the disentanglement of the *knot.* True; efforts have been tried to dissever it by the sword—not of *reason* and *science,* but of *fancy* and *conjecture;* and the blows have but rebounded on the feeble pretenders and aspirants who unskilfully dealt them.

Somewhat more we believe than three centuries ago, Mexico and Peru were found by two bands of European rovers, in the singular, not to call it the *marvellous* condition to which we have referred. They were two populous and apparently powerful empires, under the restraint of discipline and law, and not a little advanced in civilization, wealth and science, luxury and the arts. Yet they had but little, if any, intercourse with one another, and none with any other civilized people, and were situated like two vast islands in a trackless and unexplored ocean, or two mighty oases in the midst of a boundless desert of ignorance and savagism, degradation and poverty. Nor could there be discovered, we repeat, by the ablest scheme of research that could be instituted, any adequate causes of the immense difference, in matters of mind between them and the various nations around them. In most respects the phenomenon was unique—no parallel to it then existing, or having previously existed, within the purview of history.

Greece, received much of her civilization, science, and arts from Egypt; Rome from Greece; and other parts of Europe from the Italian repositories of intellect and science, literature and taste. But for Egypt no extraneous source of instruction has yet been found—nor perhaps even fancied. Like an electron *per se*, she seems to have been to herself, from her own native endowments, the source of her own pre-eminence and grandeur.

Of Mexico and Peru the same may be affirmed. They stood alone, instructed without instructors, civilized without the influence of examples to that effect, and splendid and mighty from the working of causes inherent in themselves. Like Egypt therefore they seem to have been *originals;* not imitators, copyists, or dependents on others instead of themselves.

Such were some of the peculiarities of the Mexicans and Peruvians. But not the whole of them—nor even perhaps the most striking and unexpected. Though constituting great and independent nations, they were *no warriors*, and became the victims and slaves of a mere handful of freebooters, visiting them from a distant portion of the globe. At the head of less than two hundred Spaniards, Pizarro overthrew and reduced to the most servile condition the empire of Peru, with a population of several millions of subjects, affectionately attached to the person of their chief, and enthusiastically devoted to their religion and government. And with a Spanish band of less than five hundred, aided by auxiliaries from some of the surrounding nations, Cortez conquered and enslaved the more populous and powerful empire of Mexico—two events which, as already intimated, are uninterpreted enigmas in the history of man. To what cause or combination of causes shall we look for an explanation of the fact, that victory bound her chaplets on the brows of the few, in conflicts where their adversaries outnumbered them in the ratio of *ten thousand to one?* In such a case, had not the Mexicans and Peruvians been essentially deficient in some high qualities indispensable to success, they could, with perfect ease, have thrown themselves on their foes in numbers so overwhelming, and with a force so irresistible, as literally to tear them into fragments, or trample them under foot, and crush them in mass. Nor could any form of armour, or mode of battle have saved the invaders from such an issue. It is a question then, in anthropology, of no common interest, what were the qualities in which the South Americans were so fatally deficient? It was not in abstract personal courage. In conflicts with each other, and in wars with the surrounding nations, they not only manifested ordinary bravery, but had become the conquerors and masters of the land. It was not in personal strength and activity. In those qualities they were but little, if at all, inferior to their invaders. And the prize for which they fought was of the highest value, and the most inspiriting character, including all that is dearest in life. It was their fire-sides and their families, their altars, and the hallowed ashes of their ancestors. It was every thing that enters into the all-absorbing thoughts, and the soul-inspiring sentiments of the man and the patriot, which should render him invincible, when doing battle for his *home* and his *native land.*

Nor was it, as most writers and pretended wise-ones on the subject have contended, their vast superiority in military discipline and skill, acquired by more abundant experience in war, that rendered the few Europeans so easily triumphant over the almost innumerable hosts of Americans. Far from it. The difference in military tactics, as far as experience was concerned, between the two contending parties in Mexico and Peru, was not greater, perhaps not so great, as that which existed between the legions of Cæsar and the barbarous hordes with which he contended in Germany, Gaul, and Britain. Yet the issue of war in the two hemispheres was widely different. Notwithstanding his skill and invincible hardihood, as a soldier, and his boundless resources of mind, as a chieftain, Cæsar rarely won a cheap or an easy victory, even when the numbers he led to battle were but little surpassed by those of his enemy.

Others have attributed the easy conquest of the Mexicans and Peruvians to the superstitious veneration in which they held their ruthless assailants, regarding them as beings of a superior nature, who had descended to them from the skies, to become their rulers and benefactors. But that a delusion of this kind took possession of the Americans seems highly improbable. And it is still more improbable, even admitting its occurrence at the first moment of the arrival of the roving marauders, that it should have been of long duration. The inhabitants of the New World must have very soon discovered that the emigrants from the Old were as subject as themselves to bodily injuries, sickness, and other misfortunes and infirmities, and to death itself from wounds and diseases. It is even probable if not certain that, from some of these sources of calamity, especially from that of *seasoning* sickness, the strangers must have suffered much more than the natives. And if our recollection fail us not, such was actually the case. Many of the Spaniards sickened, and not a few of them died, while those whom they had reduced to bondage remained healthy. From the notion of their *divinityship* therefore, admitting it to have had an existence, the "Iberian freebooters" derived in the end but little advantage. There is reason to believe that such advantage was more than counterbalanced by the scorn which their mean cupidity, and the detestation and abhorrence which their cruelty and revolting profligacy engendered.

Still then does the question, "why were the Americans so easily subdued?" remain unanswered. And the correct answer, virtually but silently rendered in the "Crania Americana," is derived from the science of phrenology alone. They were engaged in war with a race of men *superior to themselves*—though not descended immediately from the skies. *For the Spaniards were Caucasians.* And whenever or wherever that race, which stands at the head of the great community of man, (as the nervous and cerebral tissue takes rank of the other tissues of the body,) comes into collision, whether belligerent or pacific, with either of the other races, it never fails in the end to gain and maintain a decided ascendency. To this position we confidently believe that no solid exception can be adduced from either fact or philosophy—the examples of the present, or the history of the past. Nor is it from occurrences in the New World alone that it receives at once illustration and proof. By a phenomenon of equal moment, notoriety, and interest, or rather perhaps of much greater, in the Old World, it is further and no less substantially maintained. We allude to the degraded condition in which

Hindostan, and several neighboring principalities are held by a British army, which, from its incredible inferiority in numbers to the almost boundless amount of the population it controls, might well be deemed infinitely incompetent to the mightiness of the task. That army, containing less than *eighty thousand* rank and file, not a moiety of them, we think, being natives of Europe, has already conquered, now holds in check, and virtually consigns to a degrading vassalage, one hundred and twenty millions of human beings!

To what cause or causes is this astonishing issue ascribable? The answer we think plain. The Asiatics, though not all of a really different *race* from the Europeans who enslave them, are a *degenerate*, perhaps a mongrel branch of the same race. They are not genuine Caucasians; while a large portion of their conquerors and masters, are Anglo-Saxons—that variety which stands decidedly at the head of the Caucasians, and is their highest caste.

Are we asked to specify the actual difference between the Anglo-Saxon and the Hindostanic varieties of man, which gives to the former such a marked superiority over the latter? We reply that it consists in the different size and form of the brain in those varieties, which are fully disclosed, by corresponding difference in the size and form of their "crania." Not only is the *entire* brain of the Anglo-Saxon considerably larger than the brain of the Hindoo; the superior and truly governing organs of it are larger in a still greater proportion. Are we again asked to name those ruling and power-bestowing organs? They are, we reply, more especially Combativeness, Destructiveness, Self esteem, Approbativeness, Frmness, and the *reflecting* organs. All the organs calculated to give greater strength and energy of character, and greater scope and vigour of thought, are larger. Hence the native and necessary superiority of the Caucasians, especially of the Anglo-Saxon branch, in war, as well as in the higher walks of science, literature, and the arts.

For the easy conquests of Mexico and Peru, similar causes may be correctly assigned. Those events were attributable, not to any superiority of civilization and education, on the part of the *invaders*. In these points the *invaded* were nearly, if not quite on an equality with them. The cause was to be found exclusively in the superiority of native strength and compass of mind on the part of the Europeans. And those qualities arose from the greater size, and better development and configuration of their brains.

The Spaniards were a branch of the Caucasian race. And though they did not belong to the most highly gifted and most efficient branch, they were greatly superior to the American race, with whom they were in conflict. And that superiority was indicated by the greater size, and better development and shape of their crania and brains. They had, in their cerebral organization, a larger endowment of ground for intellectual qualifications, and comparatively a less preponderance of that forming the seat of mere animality. And these native advantages of brain bestowed on them a range and measure of mental compass and power, which the inferiorly organized, and weaker-minded Americans were unable to resist. Those comparative *imbeciles* stood related to their *vigorous assailants*, as boys do to men, idiots to sound-minded persons, or as inferior animals to the human race. Hence the amazing suddenness and completion of their overthrow and degradation!

Groundless and visionary as this position will no doubt appear to those who have *never made the subject of it a matter of study*, it will present itself in that light to *such persons only.*

Individuals sufficiently acquainted with it will view it very differently. They will regard it as one of the most grand and impressive physiological truths that has ever been disclosed. For physiological the phenomena it relates to are—as clearly and decidedly so, as the digestion of food, the secretion of bile, or the circulation of the blood. Yet was it never dreamt of as such until the discoveries of Gall, which will yet be acknowledged to constitute themselves one of the chief scientific triumphs of the nineteenth century; while their fruits will be deposited in the temple of Philosophy, among the most glorious and invaluable trophies her ministers have won.

Nor is it the so deemed mysterious events of the conquest of Mexico, Peru, and Hindostan, that the discoveries of the great German are destined to illuminate and make intelligible and useful. They will render to mankind a similar service, as relates to many other enigmas that have confounded the anthropologist and eluded his scrutiny. In truth they will yet be referred to, by the students and masters of mental and moral science, as the great expounders of the philosophy of history. They will shed on the deeds and characters of the ancient Greeks a light which the world has never yet enjoyed. They will disclose the causes of the ambition, wars, and conquests of Philip and Alexander. They will tell why Cæsar first glorified and then enslaved his country, and ultimately fell by the dagger of Brutus; why the Roman empire, after having become and continued for centuries, a marvel of power and greatness, injustice and crime, was reduced at length to a mighty ruin, by barbarian invaders; why Palestine was inundated by the mingled blood of the Crusaders and the Saracens; why the clouds of the Dark Ages, brought down on the world by the disasters of the sword, were ultimately dispersed by the return of the sun of literature and science; why Napoleon first astonished the world by the miracles of his greatness and power, and then ended his career in captivity and exile ; and why our own country was rendered independent and glorious, by Washington and his compatriots ; and has increased in wealth and renown, and their concomitants, with a rapidity and steadiness altogether unprecedented in the annals of nations.

These were all physiological events, produced through the functions of the human brain, and will hereafter be universally acknowledged as such, by those who shall become competent judges of the subject. And for this great result, the world will be indebted to the genius and labors of the founders of phrenology. Physiologists and philosophers will learn and acknowledge, that man, to be studied correctly, as a being to be acted on mentally himself, or to act by mind on others, whether for elevation or degradation, for good or for evil, *must be studied through his brain.* And that in all their manifestations and conditions, his moral and intellectual natures, instead of being any longer investigated by or through the laws, supposed to regulate abstract spirit, must be approached and comprehended (if comprehended at all,) through the instrumentality of the material machinery, on which his spirit immediately acts. In other words ; that all the events and phenomena, in which man is concerned, either as agent or subject, and whether they be peaceful or belligerent, scientific or literary, instead of being regarded, as heretofore, as the *immediate* products of mind, will be considered, in time to come, as referable to mind, only through the attributes of the nervous system. Thus will anatomy and physiology be justly ranked among the most elevated branches of human knowledge, and be received and recognized as the true foundation of anthropology and mental philosophy.

This digression however we must pursue no further, but here conclude both it and our review. Meantime it is our purpose to bring the "*Crania Americana*" once more before the public, in a future number of the Journal, when we shall endeavor to submit to our readers a fuller, more direct, and more detailed analysis of it, than we have given in the present.

